# Individual and population dietary specialization decline in fin whales during a period of ecosystem shift

**DOI:** 10.1038/s41598-021-96283-x

**Published:** 2021-08-25

**Authors:** Cabrol Jory, Véronique Lesage, Alexandra Leclerc, Janie Giard, Sara Iverson, Martine Bérubé, Robert Michaud, Christian Nozais

**Affiliations:** 1 Maurice Lamontagne Institute, Fisheries and Oceans Canada, Mont-Joli, QC Canada; 2 Département de Biologie, Chimie et Géographie, Québec-Océan, Université du Québec à Rimouski, Rimouski, QC Canada; 3 Groupe de Recherche et d’Éducation sur les Mammifères Marins, Tadoussac, QC Canada; 4 Department of Biology, Dalhousie University, Halifax, NS Canada; 5 Marine Evolution and Conservation, Groningen Institute of Evolutionary Life Sciences, University of Groningen, Groningen, The Netherlands; 6 Centre for Coastal Studies, Provincetown, MA USA

**Keywords:** Behavioural ecology, Conservation biology, Stable isotope analysis, Marine biology

## Abstract

This study sought to estimate the effect of an anthropogenic and climate-driven change in prey availability on the degree of individual and population specialization of a large marine predator, the fin whale (*Balaenoptera physalus*). We examined skin biopsies from 99 fin whales sampled in the St. Lawrence Estuary (Canada) over a nine year period (1998–2006) during which environmental change was documented. We analyzed stable isotope ratios in skin and fatty acid signatures in blubber samples of whales, as well as in seven potential prey species, and diet was quantitatively assessed using Bayesian isotopic models. An abrupt change in fin whale dietary niche coincided with a decrease in biomass of their predominant prey, Arctic krill (*Thysanoessa* spp.). This dietary niche widening toward generalist diets occurred in nearly 60% of sampled individuals. The fin whale population, typically composed of specialists of either krill or lipid-rich pelagic fishes, shifted toward one composed either of krill specialists or true generalists feeding on various zooplankton and fish prey. This change likely reduced intraspecific competition. In the context of the current “Atlantification” of northern water masses, our findings emphasize the importance of considering individual-specific foraging tactics and not only population or group average responses when assessing population resilience or when implementing conservation measures.

## Introduction

Understanding how dietary niche variability operates at the individual level and among conspecifics is central to defining species ecological needs, and refine conservation effort. Dietary niche varies among species largely as a result of evolutionary processes. However, optimal foraging theory and the niche concept predict feeding strategies to be continuously affected by resource availability or quality^1^. The adaptive capacity of a population to changing resources depends on the degree of individual specialization within the population^2,3^, with specialist feeders typically expected to be less adaptivethan in generalist feeders^4,5^. In generalist populations, however, the adaptive capacity will vary depending on whether the population’s dietary niche width results from generalist individuals all exploiting a broad range of prey, or from specialist individuals within the population each specializing on a different but narrow range of prey^2,3^.

A variety of factors likely influence the degree of individual specialization within a population, including intra- and interspecific competition, and food web complexity^6–8^. Individual specialization may also arise from an ecological “opportunity” created for instance, by the increase of a novel and valuable resource or conversely, by an increase in competition for a shared resource^9^. In the face of the major changes currently observed in the trophic structure of northern ecosystems with the “Atlantification” of Arctic ecosystems^10,11^, a widening of the trophic niche of the population through diet specialization of individuals on different prey might represent an efficient strategy to cope with these changes. This could be the case for long-lived predators such as baleen whales, which may go through multiple cycles of environmental variability and prey availability during their lifetime^5^.

Fin whales (*Balaenoptera physalus*) are baleen whales with a cosmopolitan distribution across major ocean regions, although they are most abundant in temperate and subpolar latitudes^11,12^. The Atlantic population is considered of particular concern in Canada, given the poor understanding of the stock structure, continued whaling in areas such as Iceland and Greenland, and local declines in abundance, including in the Gulf of St. Lawrence^12^. Despite their conservation status, knowledge about their feeding ecology is generally lacking. Compared to blue whales (*Balaenoptera musculus*) and right whales (*Eubalaena glacialis*), which are specialist feeders on euphausiids and copepods, respectively^13,14^, fin whales are considered more as a generalist predator. In the North Atlantic, fin whales are known to feed on both zooplankton and small pelagic fishes, including multiple euphausiid species, capelin (*Mallotus villosus*), Atlantic herring (*Clupea harengus*) and sandlance (*Ammodytes* spp.)^13,15–17^, although they might exploit other locally abundant prey^15,16^.

Fin whales and blue whales are sympatric in the Estuary and Gulf of St. Lawrence (EGSL), Canada, where they share euphausiids as a prey resource along with other species^16^. However, considerable inter-individual variability has been noted in the isotopic signatures of fin whales from the EGSL and other areas of the North Atlantic, raising questions about the degree of specialization and prey selectivity among conspecifics of this presumed generalist species^16,18^.

The St. Lawrence ecosystem has recently witnessed major and abrupt ecological shifts as a result of both overfishing and climate change^19,20^. Several commercial groundfish populations collapsed in the early 1990’s^21^, with a concurrent change in distribution and abundance of their prey species^22^. Additionally, oceanographic conditions, characterized by below-average seawater temperatures, changed around year 2000 towards above-average sea temperatures and below-average ice conditions, which have continued to prevail since ^20^. Populations of potential competitors of baleen whales, such as harp seals (*Pagophilus groenlandicus*) and grey seals (*Halichoerus grypus*), increased during this period^23,24^. It was suggested that these ecosystem changes caused a reduction in prey availability for baleen whales, leading to a decrease in whale abundance at some foraging sites^25^. However, how these changes in environmental conditions and trophic structure affected fin whale and other marine mammal diets remains poorly documented. Examining this question using a generalist predator with high absolute energy requirements such as fin whales is a unique opportunity to better understand individual responses to food shortages and how they cascade into population trophic niche width.

Specifically, we here examine the trophic niche specialisation of fin whales, at the individual and population levels, during a known ecosystem shift in the EGSL. While we recognize that fin whales sampled in the St. Lawrence Estuary during our study represent only a segment of the North Atlantic fin whale population, the term population is used hereafter for simplicity, when comparing dietary niche between individuals and our group of sampled individuals. We examine various niche metrics, as well as diet composition and degree of individual specialization. Specifically, we test the hypothesis of a broadening of fin whale dietary niche both at the individual and population level in response to the abrupt change in the St. Lawrence ecosystem structure, and associated prey availability around year 2000. To do so, we used a combination of stable isotope (δ^13^C and δ^15^N) and fatty acid (FA) trophic markers analyzed from fin whale skin and blubber biopsies (n = 99) sampled between 1998 and 2006, along with that of potential prey species. Our results provide important information about the expected consequences of the current changes observed in the St. Lawrence and other northern latitude ecosystems on fin whales and other large marine predators, and crucial information relevant for implementation of efficient conservation measures.

## Results

### Fatty acid and isotopic signatures of potential prey

**Figure 1:**
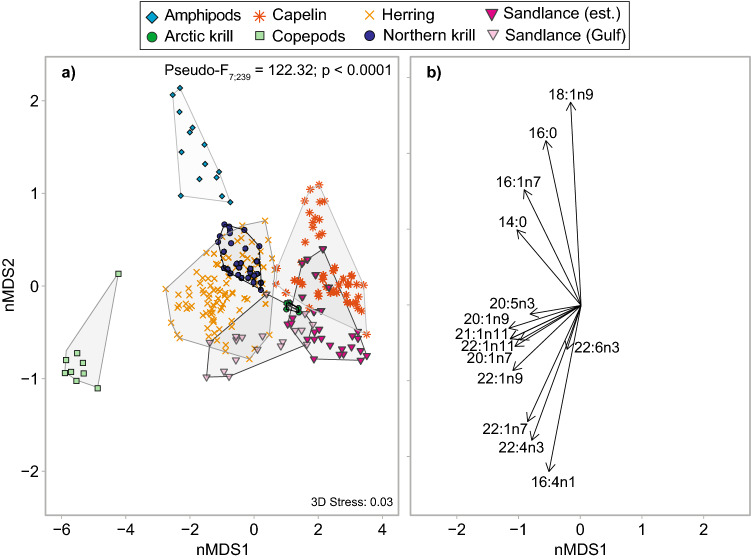
Multidimensional scaling (n-MDS) ordination in Euclidean space of **(a)** the fatty acid signature of potential prey sampled in the Estuary and Gulf of St. Lawrence between 1999 and 2005, and **(b)** fatty acids responsible for these groupings (Pearson correlations). Calculations were based on the extended dietary fatty acid subset (n = 39). Detailed interactive and static 3D n-MDS is also available in the Supplementary Fig. S1 online and in Mendeley data repository (https://doi.org/10.17632/6nxhjx9gbw.1).

The seven potential prey sampled in the EGSL differed significantly in FA composition using the extended dietary FA subset (PAIRWISE; all P < 0.004; Fig. 1a and Supplementary Table S1 online). Overall, 14 FAs were responsible for more than 70% of the dissimilarity among potential prey species (SIMPER analyses; Fig. 1b). The Σ20:1, Σ22:1 and 16:1n7 FA isomers largely differentiated copepods from the rest of the prey species. These FAs, along with 14:0, also contributed to separating capelin from herring (up to 60% according to SIMPER analyses). These FAs, combined with 18:1n9, 16:4n1, 20:5n3, and 22:6n3, differentiated the two krill species and further differentiated krill species, and krill as a group from amphipods (Fig. 1 and Supplementary Table S1 online). We also noted a statistically significant difference in the FA composition of sandlance depending on sampling region (PERMANOVA; *Pseudo-F*_*1*;46_ = , P < 0.0001): concentrations of Σ20:1, Σ22:1, 20:5n3, and 22:6n3 were higher, and concentrations of 16:0 and 18:1n9 were lower, in sandlance sampled in the Gulf compared to the Estuary (Table S1).

The seven prey species also differed to some extent in their stable isotope ratios. However, Arctic krill and calanoid copepods were isotopically indistinguishable (PAIRWISE, P = 0.68), and were therefore considered a functional group for diet estimations. The same pattern was observed for capelin and herring (PAIRWISE, P = 0.92). While the two species were aggregated when included in isotope mixing models, capelin/herring from the Estuary were considered separately from capelin/herring from the Gulf in our analysis given the observed difference in δ^13^C values between these two regions (PAIRWISE, P = 0.002), hence the potential for this group to convey information about feeding areas.

### Isotopic and fatty acid signatures of fin whales

Overall, isotopic signatures of individual fin whales varied between −19.0 and −17.0‰ for δ^13^C, and 9.3 and 14.6‰ for δ^15^N. Male and female fin whales were similar in their isotope ratios (PERMANOVA; δ^13^C: *F*_1;97_ = 1.82; *P* = 0.19; δ^15^N: *F*_1;98_ = 3.11; *P* = 0.1) and FA composition (PERMANOVA; *Pseudo-F*_1,90_ = 0.99; P = 0.2). As a result, males and females were considered together in subsequent analyses.

**Figure 2:**
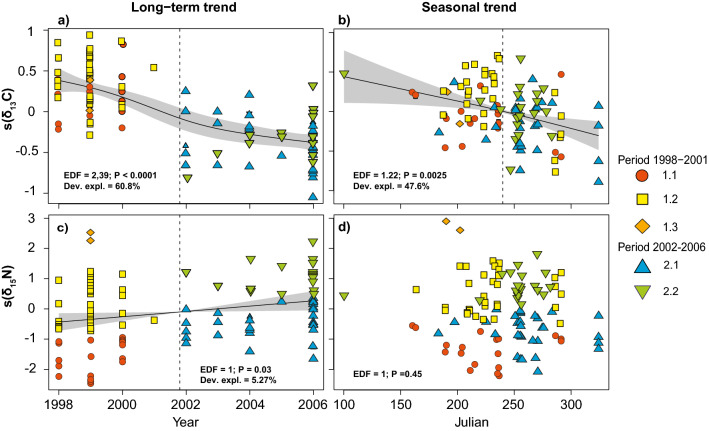
Long-term **(a,c)** and seasonal trend **(b,d)** in δ^13^C and δ^15^N values for fin whales sampled in the St. Lawrence Estuary between 1998 and 2006, superimposed with groups of fin whales as defined from the hierarchical cluster analysis (different symbols and colors). The y-axis shows deviations from mean isotopic values; shaded areas represent the 95% credibility interval around the generalized additive model; the dotted line represents the time of observed changes.

Both seasonal and long-term trends were observed in stable isotope ratios (Fig. 2). Lower δ^13^C values were observed in both late summer (after August 20^th^), and beginning in 2002 and later years (Fig. 2a,b). This pattern was unrelated to potential sampling biases as indicated by the persistence of the significant temporal trend over the time series after controlling for year effect (seasonal trend) or when considering the two seasons (summer and fall) separately (yearly trend). No seasonal trend was observed in nitrogen, but δ^15^N values increased linearly over the study period 1998–2006 (Fig. 2c).

**Figure 3:**
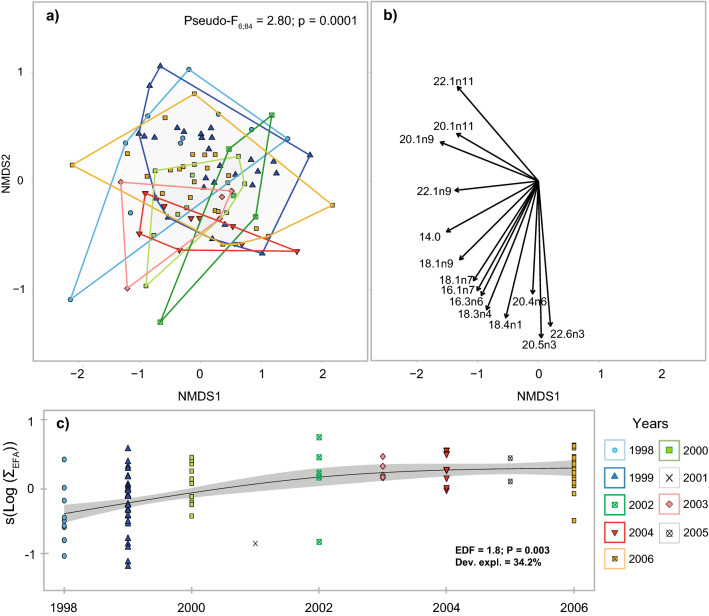
Multidimensional scaling (n-MDS) ordination in Euclidean space of **(a)** the fatty acid composition of fin whales, **(b)** fatty acids responsible for these groupings (Pearson correlations), and **(c)** long-term trend in the proportion of major essential fatty acids (Sum of 20:5n3, 22:6n3 and 20:4n6). nMDS calculations were based on the extended dietary fatty acid subset (n = 39). Avg. = Population average. Detailed interactive and static 3D n-MDS is also available in the Supplementary Fig. S2 online and in Mendeley data repository (https://doi.org/10.17632/6nxhjx9gbw.1).

FAs in fin whale blubber did not change seasonally (PERMANOVA; *Pseudo-F*_*2*;89_ = 1.91; P = 0.08), but varied inter-annually (Fig. 3). More than 70% of these variations were explained by 14 FAs (SIMPER analyses; Fig. 3b). In addition, major essential FAs, represented by the sums of 20:5n3, 22:6n3 and 20:4n6, increased between 1998 and 2003, thereafter remaining constant until at least 2006 (Fig. 3c).

### Diet estimation and specialization at the population level determined from isotopic mixing models

**Figure 4:**
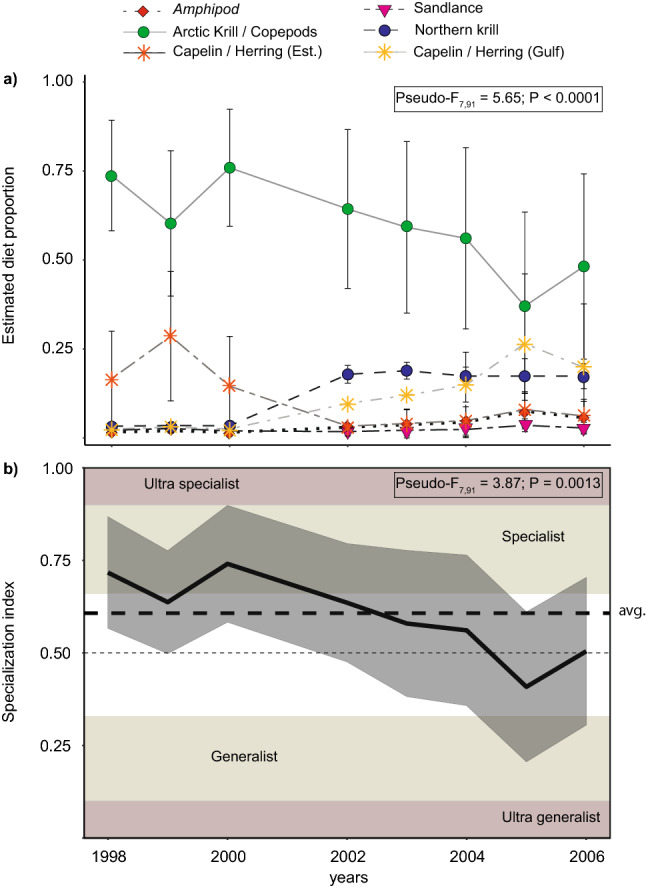
Inter-annual variation of **(a)** the estimated proportional contributions of each potential prey to fin whale diet from isotopic mixing models, with **(b)** the corresponding specialization index Ɛ over the study period (1998–2006). *Avg.* population average.

The isotopic mixing models estimated a diet for fin whales composed predominantly of euphausiids when integrated over the entire study period 1998–2006 (Fig. 4a). Together, northern krill and the functional group Arctic krill/copepods represented 69.8 ± 23.7% of the estimated fin whale diet, with capelin/herring (24.6 ± 20.4%), amphipods (3.1 ± 3.1%) and sandlance (2.4 ± 1.7%) accounting for diminishing proportions of the diet. Before 2002, 89.5 ± 2.5% of the estimated diet was dominated by Arctic krill/copepods and, to a lower extent, pelagic fish (capelin/herring) from the St. Lawrence Estuary. During this period, the proportion of pelagic fish from the Estuary was times higher than pelagic fish from the Gulf in fin whale diets (Fig. 4a). In 2002 and subsequent years, fin whales appeared to feed to a lower extent on Arctic krill/copepods and capelin/herring from the Estuary (together 56.9 ± 19.8%), and incorporated northern krill and to a lesser extent amphipods as other prey in their diet. It was also estimated that whales consumed a higher proportion of capelin/herring from the Gulf during 2002–2006 compared to previous years (Fig. 4a). As a result, there was a significant change in the specialization index of fin whales over the study period (Fig. 4b). Dietary habits shifted around 2002 from diets that were more typical of specialist feeders with Ɛ varying from 0.51 to 0.89 (Ɛ_mean_ = 0.68), to some that included true generalist feeders (Ɛ_min_ = 0.21).

### Individual diet specialization

Given the observed shift in fin whale isotopic signatures starting in 2002 (Fig. 4), a pre- and post-2002 periods were considered in the cluster analysis for identifying natural groupings among individuals. This analysis revealed five isotopically distinct groups among the sampled fin whales, including three groups for the period 1998–2001 and two groups for 2002–2006.

**Figure 5:**
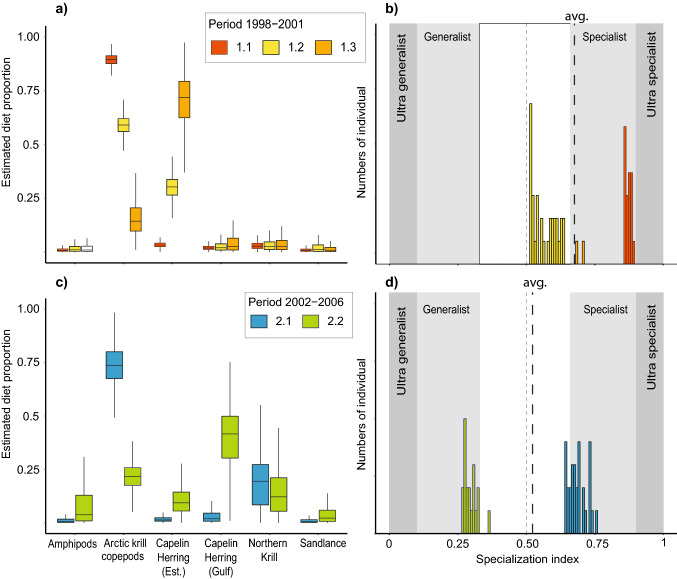
Estimated proportional contributions of potential prey determined from isotopic mixing models **(a,c)** and specialization index frequency **(b,d)** for each group of fin whales identified through the hierarchical cluster analysis for the periods 1998–2000 **(a,b)** and 2002–2006 **(c,d)**.

Before 2002, two of the three groups (1.1 and 1.3) were highly selective, either toward Arctic krill/copepods (Group 1.1) or capelin/herring (Group 1.3), which accounted for an average of 89.2 ± 2.7% and 78.1% ± 22.1% of their diet, respectively (Fig. 5a). Group 1.2, which represented the majority of the individuals sampled before 2002 (~ 60%), adhered more to a “specialist-generalist” feeding behavior (0.5 < Ɛ < 0.6; Fig. 5b) with a diet composed of Arctic krill/copepods and capelin/herring in almost similar proportions (Fig. 5a).

In 2002 and subsequent years, 40% of the sampled individuals (Group 2.1) selected almost exclusively zooplankton, which contributed > 90% to their diet. Individuals from this group were estimated to feed on both Arctic krill/copepods and Northern krill as well as on amphipods, thus also adhering to a “specialist-generalist” feeding behavior similar to fin whales from the earlier period (Ɛ ~ 0.55; Fig. 5c). However, the other 60% of the population (Group 2.2) exhibited a generalist feeding behavior (Ɛ < 0.34) by consuming prey from all functional groups, although with a higher representation of pelagic fish in their diet (Fig. 5c,d).

## Discussion

This study reported on novel aspects of the foraging ecology of North Atlantic fin whales. There was a marked inter-individual variability in dietary habits and a widening of fin whales’ trophic niche both at the population and individual level during a period of documented environmental change in the EGSL. These changes were concomitant to a decrease in biomass of the whales’ primary prey, and resulted in an apparent shift in feeding areas for individuals targeting pelagic fish. Together, these findings add a new level of complexity for the conservation of this population of special concern in Canada^12^.

Isotopic mixing models indicated that fin whales using the St. Lawrence estuary as part of their feeding habitat consumed predominantly Arctic krill/copepods in 1998–2006, with capelin/herring representing about 20% of their diet. Copepods are likely unimportant in the diet of fin whales^17^, leaving Arctic krill as the primary contributor of this functional group to the diet. Arctic krill is also an important prey for fin whales feeding in the Gulf of St. Lawrence, representing on average 56% of the diet in 1992–2010^16^. The relative dietary contributions of capelin versus Atlantic herring could not be assessed from stable isotopes due to their similarity in isotopic composition, nor from their FA signatures due to limitations imposed on quantitative analyses when calibration coefficients are unknown^26,27^. Furthermore, information on the abundance of different prey species is fragmentary for the EGSL, preventing speculation about their relative availability to fin whales both spatially and seasonally. Both capelin and herring are lipid-rich species (Table 1) and known prey of fin whales elsewhere^16,18,28^. They are sympatric and available throughout the feeding season (i.e., from spring to fall) in the EGSL, although their relative availability may vary at finer scales within these regions^29^.

**Table 1:** Sample size, descriptive characteristics and mean δ^13^C and δ^15^N values for fin whale potential prey collected between 1999 and 2005 in the Estuary and Gulf of St. Lawrence and analysed for stable isotope (SI) or fatty acid (FA) analyses.

Potential prey	Sampling region		N_tot_	Years							Length (cm)	Wet weight (g)	δ^13^C ± SD (‰)	δ^15^N ± SD (‰)	C:N ratio	% lipid
				1999	2000	2001	2002	2003	2004	2005						
Sand lance (*Ammodytes* spp.)	Est	SI	21	–	–	15	–	–	6	–	12.1 ± 4.1	5.4 ± 6.9	−18.7 ± 0.5	10.8 ± 0.3	3.16 ± 0.05	2.9 ± 1.7
		FA	18	–	–	6	6	6	–	–						
	Gulf	SI	6	–	–	5	–	–	1	–	12.7 ± 2.3	6.2 ± 3.0	−18.8 ± 0.5	10.9 ± 0.4	3.17 ± 0.02	3.7 ± 1.8
		FA	25	–	20	5	–	–	–	–						
Copepods *(Calanus* spp.)	Est	SI	42	–	–	32	10	–	–	–	–	0.12 ± 0.05	−18.4 ± 0.9	9.3 ± 0.4	3.25 ± 0.13	16.5 ± 3.6
		FA	10	–	–	10	–	–	–	–						
Herring (*Clupea harengus*)	Est	SI	40	–	–	30	–	10	–	–	23.1 ± 5.5	122.4 ± 69.8	−17.9 ± 0.9	12.7 ± 0.6	3.12 ± 0.06	7.1 ± 3.5
		FA	96	–	15	55	11	15	–	–						
	Gulf	SI	10	–	–	–	–	8	2	–	9.5 ± 3.3	6.3 ± 6.1	−19.6 ± 0.3	12.2 ± 0.5	3.22 ± 0.08	–
		FA	**–**	–	–	–	–	–	-	–						
Capelin (*Mallotus villosus*)	Est	SI	100	–	–	–	–	56	4	40	12.8 ± 2.2	14.4 ± 7.0	−18.7 ± 0.3	12.4 ± 0.4	3.16 ± 0.03	2.8 ± 3.0
		FA	43	–	10	33	–	–	–	–						
	Gulf	SI	10	–	–	–	–	1	–	9	10.8 ± 0.7	5.3 ± 1.3	−19.5 ± 0.2	12.1 ± 0.4	3.14 ± 0.04	–
		FA	–	–	–	–	–	–	–	–						
Northern Krill (*Meganyctiphanes norvegica*)	Est	SI	110	–	–	54	18	35	3	–	–	0.83 ± 1.38	−19.5 ± 0.5	10.7 ± 0.4	3.2 ± 0.05	3.2 ± 1.0
		FA	28	10	–	10	2	6	–	–						
Amphipods (*Themisto* spp.)	Est	SI	34	–	–	0	13	18	3	–	–	0.94 ± 0.37	−19.2 ± 0.4	11.9 ± 0.4	3.38 ± 0.1	4.2 ± 1.5
		FA	14	–	–	7	3	4	–	–						
Arctic krill *(Thysanoessa* spp.)	Est	SI	28	–	–	8	–	15	5	–	–	1.57 ± 2.81	−18.7 ± 0.3	9.3 ± 0.6	3.23 ± 0.08	6.5 ± 0.8
		FA	5	–	–	5	–	-	–	–						

Central tendency is expressed as the mean (± SD). Isotopic values are uncorrected for isotopic discrimination, but are corrected for lipid extraction.

Similarly, little information exists about the abundance of sandlance in the EGSL where it is suspected to be abundant^29^. This species may be an important prey for a number of marine mammal species in the St. Lawrence Estuary such as the beluga (*Delphinapterus leucas*)^30^ and possibly also for fin whales as previously suggested for the Gulf of St. Lawrence^16^. In the latter study, also based on stable isotope analysis, sandlance occupied a functional group with northern krill that was estimated to contribute up to 50% of fin whale diets^16^. While the relative contribution of sandlance and northern krill to fin whale diet in the Gulf remains unclear, our results indicate that northern krill may be a more important prey item than sandlance, with contributions varying among individuals from 0.9–56.4% for northern krill compared to 0–11.2% for sandlance.

Stable isotope ratios measured in the skin likely integrate diet over two to three months in odontocetes^31^. Although no value exists for the skin of larger cetaceans, the turnover rate has been suggested to be lower in baleen whales compared to toothed whales^32^. Accordingly, the skin of fin whales sampled in the early/middle of summer should reflect their average diet since early spring, and those sampled in the fall should reflect more of their summer-early fall diet. A progressive depletion in ^13^C occurred from June to November, with no change detected in trophic position (nitrogen isotope ratios; Fig. 2) after controlling for year effects. These results are consistent with other dietary studies of fin whales in the North Atlantic, which indicate a shift from krill-dominated diets during summer to fish-dominated diets in the spring and/or fall^33^. However, they contrast with a previous isotopic study of fin whales sampled in the Gulf of St. Lawrence which suggests constancy in diet across seasons^16^. The latter study however, was conducted in what is known as the main aggregation area for fin whales in the St. Lawrence (Estuary or Gulf). Fin whales from that feeding location might be less likely to move elsewhere compared to whales occupying secondary feeding areas such as the Estuary^12,34^. The progressive carbon depletion that was observed over the season in our study probably reflects a change in diet and/or a shift in feeding area toward the more ^13^C-depleted Gulf of St. Lawrence^35^, as fin whales increased their intake of northern krill or capelin/herring from the Gulf of St. Lawrence.

Overall, these results confirm previous findings that identified fin whales, when studied at the population level, as relatively generalist species feeding partly on krill and partly on small pelagic fishes in various environments, including the EGSL^16^, the northern and eastern Atlantic^28,36^, the Pacific^37^ and the Mediterranean Sea^38^. However, our diet estimation and specialization indices revealed the existence of two contrasting foraging strategies among fin whales using the St. Lawrence Estuary as part of their foraging habitat, with the coexistence of highly specialized individuals, as well as true generalist individuals. Our results also highlighted a partitioning of food resources among the specialists (i.e., Ɛ < 0.7), with individuals feeding almost exclusively (up to 80% of the diet) on Arctic krill or, for a smaller number of individuals, almost exclusively on lipid-rich pelagic fishes (capelin/herring). These results, combined with those obtained from fin whales in the Gulf of St. Lawrence^16^ highlight the importance of these resources, especially Arctic krill, in these environments. The diversity observed in the diet composition among individuals also suggests that some degree of competition may occur for food resources.

Intraspecific competition is considered one of the primary drivers of individual specialization, and could be even more important than interspecific competition for shaping individual behaviour within a population^6,39^. By reducing prey availability and energy return over investment, intraspecific competition may lead to a specialization on different prey types, reducing the potential for competition to only a subset of the population^2^. Within baleen whales and probably also for other taxonomic groups, specialist feeders may also become particularly efficient at exploiting a specific resource compared to a generalist feeder by developing manoeuvers that enhance feeding success^40^, or through increased knowledge of areas and times of abundance of specific prey^41^. The importance of landscape (or seascape for marine mammals) knowledge and social interactions in explaining resource use variations has been the focus of multiple recent studies in both terrestrial and marine mammals^42,43^. However, such variation arising from phenotypical traits may not be pronounced in a low-competition environment, and may emerge only when intraspecific competition for preferred resources intensifies^6^ . Whether the observed differences in diet and degree of specialty among fin whales in our study resulted from particular functional traits is unknown given the lack of information about age, site fidelity, or stability of individual diets through time.

A reduction in prey availability may lead to increased specialization, or alternatively to diversification of diet, in both cases resulting in a widening of the population’s trophic niche^3^. Morphological, behavioral, and physiological traits can affect the capacity of individuals to use different resources^7^. Fin whales are filter feeders that engulf large prey/water quantities during lunges^44^. This feeding strategy requires a high degree of physiological adaptation and is energetically costly, so it is efficient only with schooling pelagic prey that have limited escape capacities^45^. This likely limits niche expansion of fin whales toward diets that include non-gregarious, larger prey. The slight increase observed in nitrogen isotopic ratios of fin whales over the course of the study period may reflect this limitation on ingestion of larger prey, but also the inclusion of new zooplankton species from slightly higher trophic positions (Fig. 2). Niche expansion through diet diversification is also constrained by interspecific competition^46^. Individual niche expansion through the addition of new resources will only be beneficial if additional costs incurred from the interspecific competition are lower than the energy gains provided by exploiting new resources (e.g., decrease of intraspecific competition).

The shift we observed in fin whale diet occurred during a period of documented environmental change in the EGSL^22^, which is suggested to have reduced prey availability, and enhanced the potential for intraspecific and interspecific competition. During this period, demographic changes were noted in a number of marine mammal species, which might have direct bearing on intra- and interspecific competition. In the case of fin whales, an increase in intraspecific competition as a direct consequence of an increase in fin whale abundance over the study period was deemed unlikely given that their numbers have been declining since the early 2000s in the Gulf of St. Lawrence ^34,47^. However, intraspecific competition may have arisen from a reduction in the number or size of prey patches, and an increased competition for the same space/resources. Several species of marine mammals, seabirds and fish are also known consumers of some of the forage species targeted by fin whales^19^. Species such as harp seals and grey seals have substantially increased in abundance over the past decades^23,24^.However, the degree of interspecific competition for prey resources during the study period remains largely unknown, and may require further consideration in future studies.

The coincidence between the observed shift in fin whale diet and the change in preservation method (from DMSO to deep-freeze) was noted and closely examined. The correction factors for this effect were derived specifically for fin whales and other Balaenopteridae^48^. In order for the results to be a total artifact of the methodology, they would need to be systematically biased upwards in the case of carbon isotopic ratios and downward in the case of nitrogen isotopic ratios. However, our experimental studies based on 24 individuals where pairs of samples were preserved under both conditions, did not produce a mean under-correction for ^13^C, and a mean over-correction for ^15^N. While the application of a correction factor might have increased variability in the results, we believe that a directional bias in our corrections remains unlikely.

The diversification of fin whale diets after 2001 likely occurred in response to a decrease in Arctic krill abundance or availability during this period of warming oceanographic conditions^20^ and community changes^45^. Krill species in the EGSL are composed of two genera exhibiting distinct thermal niches^50^ and ecology^51,52^. While total krill biomass changed little over our study period, krill composition shifted progressively toward a dominance of the more temperate species, the northern krill^49^. From 1999 to 2008, the biomass of Arctic krill declined by almost 2.5-fold, with egg production by this species dropping from > 90% to ~ 40% of total production^49^. Northern krill is three times larger than Arctic krill but has lower lipid contents and forms more diffuse and deeper swarms^53^, affecting both the quality and the quantity of energy transferred to consumers.

Little is known about the suitability of the Estuary or Gulf of St. Lawrence as a foraging ground for fin whales or other rorquals during the 1990s or 2000s compared to earlier or later years. Nonetheless, a recent bioenergetic study of a close relative—the blue whale—with similar biomechanics^45^, concluded that feeding in the EGSL may lead to a positive energy balance for blue whales, and that the potential for energy gain was higher when feeding on northern krill compared to Arctic krill, regardless of feeding depth^54^. However, densities of northern krill that are worth exploiting by fin or blue whales might be less frequently encountered than for Arctic krill, even during the warming period, reducing the overall benefit of feeding on northern krill in the EGSL^55^. According to optimal foraging theory, an individual should aim at maximizing net energy intake in a relatively predictable environment by limiting search time on less valuable species^56^. Accordingly, fin whales can alter diet composition or move among foraging habitats to meet their energetic needs. Changes in the marine environment at the turn of the twenty-first century likely reduced the predictability of fin whale foraging habitat, resulting in a modification of their realized trophic niches both at the individual and population level, and in an apparent change in the relative use of the EGSL for foraging. While the time spent feeding in the EGSL cannot be ascertained, our diet estimates indicate a major increase in the proportion of capelin/herring from the Gulf of St. Lawrence in their diet in 2002 and following years, suggesting a more extensive use of the Gulf at that period. A similar decrease in the use of the St. Lawrence Estuary has also been documented in foraging blue whales, a krill specialist^16,20^. Together, these observations suggest a decline in the quality of the foraging habitat for fin and blue whales. The decline in fin whale abundance in the Gulf of St. Lawrence starting in the early 2000s, which was exacerbated starting around 2010 and associated with a decline in krill biomasses ^57^, supports this hypothesis. Year 2010 marked the beginning of extremes in warmth and light ice conditions in the EGSL and other ecosystems of the North Atlantic^20^. These changes in ecosystem conditions coincided with changes in reproduction, body condition, survival rates, or distribution not only of fin whales, but of a number of other marine mammal species including St. Lawrence Estuary beluga, humpback whales, and North Atlantic right whales ^47,57–59^.

As capital breeders, fin whales rely largely on endogenous reserves accumulated during the feeding season for fueling the costs of reproduction and calving. For these species, the quantity and quality of food resources are determinant of the energy surplus accumulated and can greatly influence their fitness^12^. The decline observed in the abundance of fin whales in the EGSL since the early 2000s, which was also accompanied by lower recruitments and survival, support our conclusions about feeding conditions after 2001 being generally unfavorable for fin whales, and persisting beyond our study period^34,57^. Fin whales accumulate energy reserves as lipid stored primarily in their blubber layer in the form of FAs assembled in triacylglycerol. A recent study in St. Lawrence Estuary beluga indicates that the absolute abundance of essential FAs can be used as a proxy for body condition^58^. Our study indicates a progressive increase in the relative abundance of major essential FAs from 1998 to 2000, and leveling off over the rest of our study period until 2006 (Fig. 3c). The lack of information about lipid contents in our study precluded us from determining whether this trend was related to changes in the abundance of specific FAs and thus, whether fin whale body condition changed over time. Further sampling of fin whales in more recent years would help determine whether the changes in habitat use and vital rates documented recently in the segment of the fin whale population using the St. Lawrence have physiological foundations^57^, as recently demonstrated for St. Lawrence Estuary beluga^58^.

To conclude, using stable isotope and FAs analyses, we highlight the importance of going beyond population mean diets to understand population dynamics of generalist feeders, and their response to environmental variability. In the context of the rapidly changing climate and the uncertainty about the distribution range and population structure of fin whales in the western North Atlantic^20^, our findings underscore the need for further research on current population-level and individual trophic niche and habitat use, and the importance of accounting for interindividual variability in feeding strategies when implementing conservation measures.

## Methodology

### Field sampling

**Figure 6:**
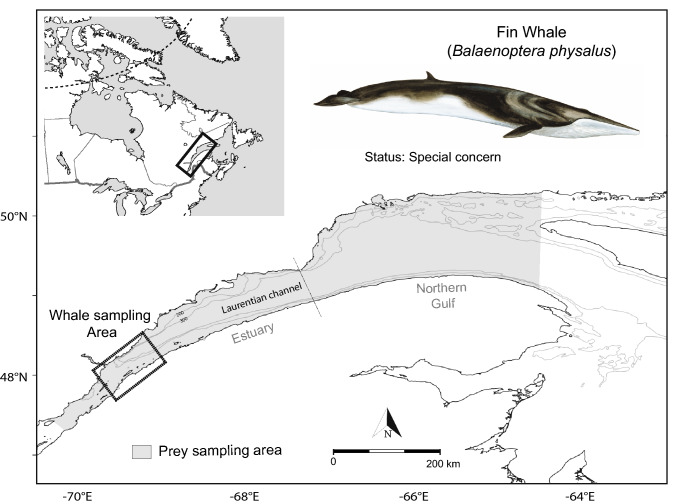
Sampling area where fin whales (black square) and potential prey (shade area) were sampled between 1998 and 2006. The dashed line indicates the boundary between the Estuary and Gulf of St. Lawrence. Map was produced by the authors with R (v3.6.2, https://www.r-project.org/) using a bathymetry data provided by the Canadian Hydrographic Service. Fin whale drawing was kindly provided by R. Michaud.

We collected a total of 114 biopsies from 99 fin whales sampled over a nine-year period (1998–2006) in the St. Lawrence Estuary, Canada, during June to November when they and other marine mammal species aggregate to feed intensively (Fig. 6)^60^. We identified individuals using pigmentation patterns, scars, and shape of the dorsal fin^61^, except for a few individuals (~ 8%) where pictures were not available or were of insufficient quality. For individuals known to have been sampled twice in a given year, only one randomly-selected biopsy was used for analyses to avoid pseudo-replication. The few individuals that were resampled two (n = 6) or three times (n = 1) over the study period were included in the analyses. Sex was determined genetically^62^ and revealed a similar proportion of males and females in our sample (47.5% and 52.5%, respectively).

We obtained biopsies by remotely projecting a dart (40 mm in length and 8 mm in diameter) from a 5–8 m vessel. Given the thickness of the skin and blubber layer in fin whales (5 to 25 cm depending on location on the body^63,64^), blubber samples likely included only the outer blubber layer. From 1998 to 2001, the epidermis was separated from the blubber and preserved in a dimethyl sulfoxide solution (DMSO, 20% v/v) with deionized water saturated with NaCl, frozen at − 20 °C, while blubber was frozen directly at − 20 °C. Starting in 2002, both the skin and blubber were separated and frozen directly. The biopsy protocol was reviewed and approved by the Canadian Council on Animal Care committee from Simon Fraser University (Number IML-16A), and research conducted under the Fisheries Act permit DFO-QUE18-2002, R2003-003, DFO-QUE10-2004 and SAGMP-2006-738. We confirm that all sampling was performed on wild animals in accordance with these committee guidelines and regulations. In addition, we confirm that all methods are reported in accordance with ARRIVE guidelines 2.0 (https://arriveguidelines.org).

We also sampled all potential prey species that, may be part of the fin whale diet and are locally abundant. Prey were sampled during various research cruises conducted mostly over the same period as fin whales were biopsied (April to November 1999–2005; Table 1). The prey included four zooplankton species: amphipods (*Themisto* spp.); Arctic krill (*Thysanoessa* spp); calanoid copepods (*Calanus* spp.) and northern krill (*Meganyctiphanes norvegica*), as well as three forage fish species: Atlantic herring; capelin and sandlance. When available, we selected prey from multiple locations in both the Estuary and the northern part of the Gulf of St. Lawrence (see Fig. 6) and over multiple years to account for potential movements of fin whales during the feeding season, and for spatial or inter-annual variations in prey isotopic and FA signatures (Table 1). We sampled and processed prey following methods outlined in Lesage et al.^35^. Briefly, zooplankton species were caught using Bongo nets (333 μm mesh size), while fish were captured in bottom trawls or weir nets and kept at − 20 °C until analyses.

In the laboratory, we identified, measured (± 0.1 cm; standard fork length; fish only) and weighted (± 0.1 g) individual prey items. For stable isotope analyses, we excised a small piece of muscle tissue from frozen specimens, except copepods for which the whole individual was used (N_total_ = 427, Table 1). For fatty acid analyses, we used a measured subsample of the whole homogenized specimen (N_total_ = 242, Table 1).

### Stable isotope analyses

When the chemical analyses were conducted for this study, the need to analyze aliquots separately as the best way to deal with the effect of lipid-extraction on nitrogen isotope ratios had not yet emerged from the literature ^65–67^. As a result, all whale skin and prey samples were lipid-extracted using the Folch method and a chloroform/methanol solvent mix (2:1, v/v) following the protocol outlined in Lesage et al.^35^. Isotopic signatures were lipid-corrected for this effect and that of DMSO (see Data analysis; see also Newsome et al.^67^).

Stable carbon and nitrogen isotope ratios were determined using a continuous-flow stable-isotope mass spectrometer coupled to a Carlo Erba elemental analyzer (CHNS-O EA1108). Carbonates from Vienna Pee Dee Belemnite limestone and atmospheric N were used as standards for δ^13^C and δ^15^N, respectively. Stable isotopes are expressed in δ notation as the deviation from international standards in parts per thousand (‰) according to the following equation:

1 $$\delta {\text{X }} = [\left( {{\text{Rsample}}/{\text{Rstandard}}} \right) \, {-}{ 1}] \, \times { 1}000$$

where δX is ^13^C or ^15^ N and R is the corresponding ^13^C/^12^C or ^15^ N/^14^ N ratio. Analytical error based on replicate analyses of samples and laboratory standards (n = 101) was 0.11‰ for δ^13^C and 0.12‰ for δ^15^N.

### Fatty acid analyses

Lipid was quantitatively extracted from a 1.5 g homogenate of whale blubber or homogenized prey using a modified Folch procedure following the protocol described in Budge et al.^68^. Briefly, we used a solution of 2:1 chloroform:methanol with 0.01% BHT (v/v/w) for lipid extraction and determined total lipid recovery gravimetrically after evaporation. We prepared FA methyl esters (FAME) using Hilditch reagent (0.5 N H_2_SO_4_ in methanol) and methylene chloride heated at 100 °C for 1 h. We analyzed FAMEs in duplicate using a Perkin-Elmer Autosystem II capillary gas chromatograph with silica column coated with 50% cyanopropyl polysiloxane (0.25 μm film thickness; J&W DB-23; Folsom, CA) coupled to a flame ionization detector and using Turbochrome 4 software (PE Nelson). The FAs and isomers were identified from a number of validated sources according to Iverson et al.^69^. FAs are expressed as mass percent of total FAs and described using the shorthand nomenclature of C:DnX, where C is the number of carbon atoms, D is the number of double bonds, and nX indicates the position of the double bond closest to the terminal methyl group.

### Data analyses

To account for lipid extraction and DMSO effects on δ^15^N values, we applied mathematical correction factors explicitly developed for the skin of fin whales and other *Balaenopteridae*^34^ and for the muscle of fish sampled in our study area^70^. In the absence of such correction factors for zooplankton species, and given the similarity in the C:N ratios between fish and zooplankton (see Table 1), we assumed mean enrichment of δ^15^N values as a result of lipid-extraction to be similar for zooplankton and fish^70^.

As the first step to data analysis, we examined prey stable isotope and FA signatures for potential effects of sampling location (Estuary vs Gulf) and sampling year using permutational multivariate analyses of variance (PERMANOVA)^71^. The extended dietary FAs, i.e., FAs entirely or mostly derived from diet (see Iverson et al.^27^), were used in this analysis to maximize the potential for discriminating among the different prey species. We combined prey that were similar in composition based on PERMANOVA results (and pairwise comparisons), and which shared ecological similarities^26^. We evaluated significance for all tests using the Benjamini & Yekutieli adjustment method to limit type I error and performed PERMANOVA using Euclidean distance, 9999 permutations and partial sum of squares. Results were visualized using nMDS. We identified which FAs were responsible for species differentiation using a SIMPER exploratory analysis and Pearson correlations (r > 0.6)^72^.

We used a linear mixed model (Lmer4 package) to test for potential sex effects in fin whale isotopic signatures^73^, using year and season as covariates, and δ^13^C and δ^15^N values separately. We used generalized additive models (GAMs; R package “mgcv”) to examine seasonal (Julian day) and long-term changes in isotopic signatures over our study period (1998 to 2006). Years and Julian day were included in the GAM as covariates using penalized cubic regression spline smooth. Parameters were estimated using the ‘mgcv’ package with the restricted maximum likelihood method (REML)^74^. Given the large number of FAs to consider, we used a two-way PERMANOVA (Sex x Year) to explore potential differences in FA signatures among fin whales. FAs contributing to these differences were identified using a SIMPER analysis, and were then examined for long-term trends using GAMs. All analyses were performed using the R programming language (v3.6.2, R core team 2021^75^). Data are presented as mean ± standard deviation. Fin whale and prey stable isotope and FA data are available in supplementary data online and on permanent repository (please see the section “Data accessibility” for details and link).

### Diet estimation

A mean diet estimate was calculated for fin whales over our nine-year study period using stable isotopes and mixing models (see below). However, given temporal structuring was expected in our database, we also explored natural groupings in isotopic signatures among individual whales using hierarchical cluster analysis. FAs were not included in this analysis given that their metabolism and differential deposition is poorly understood for tissues of cetaceans. We determined the adequate number of clusters from the Dunn’s index (R package “clv” version 2.2), and validated the clustering results using discriminant functions analysis (R package “MASS” version 7.3).

We then estimated diet composition using a Bayesian stable isotope mixing model (MixSIAR version 3.1.10^76^), which explicitly accounts for the uncertainty in isotopic source signatures and trophic discrimination factors (TDFs)^76,77^. There are currently no TDFs specific to fin whales that are based on controlled studies, but those that have been estimated for cetaceans vary from 0.5 to 1.3 for carbon, and 1.5–2.5 for nitrogen^16,18,78–80^. We used a median value of 1.0 ± 0.5‰ for carbon and 1.7 ± 0.5‰ for nitrogen; the large standard deviations associated with the TDFs are meant to recognize the current uncertainty in these values. In addition, the proposed TDFs are also consistent with the 1.3—1.9‰ TDF value for ∆^15^ N predicted by Caut et al.^81^ based on the isotopic value of fin whale hypothetical prey. We used a non-informative Dirichlet prior distribution and no concentration dependencies. The model was set as “extreme” with process error only and using groups as a fixed effect and individuals as a random effect^82^. Gelman-Rubin and Geweke metrics confirmed model convergence.

While estimated TDFs are available for stable isotopes, calibration coefficients for the predictable deposition of FAs into the blubber of cetaceans are largely unknown, limiting the use of quantitative FA signature analysis (QFASA) for diet quantification in these taxa^65^. As a result, diet based on FA composition (using the extended FA subset^27^) were only qualitative in this study^68^. Interannual, seasonal and sex-specific patterns were examined using PERMANOVA, and the FAs responsible for these variations were visualized using ordination methods.

### Individual specialization

A specialization index (Ɛ)^83^ was estimated based on compositional diet estimates obtained from isotopic mixing models^84,85^. Briefly, Ɛ was calculated as the distance between a hypothetical diet vector of an ultra-generalist consumer (i.e., feeding equally on all prey resources) and the estimated diet for each fin whale in an Euclidean space. The specialization index varied between 0 and 1, where 0 corresponded to an ultra-generalist and 1, to an ultra-specialist diet. Specialization indices are presented as means ± associated standard deviations (SD).

## Supplementary Information

Supplementary Information 1.

Supplementary Information 2.

Supplementary Information 3.

Supplementary Information 4.

## Data Availability

All datasets (i.e., isotopic and fatty acid signatures) that support the findings of this study as well as supplementary figures and tables are available online as supplemental data, in the following Mendeley data repository (https://doi.org/10.17632/6nxhjx9gbw.1) or from the corresponding author upon reasonable request.
