# Supplementary figures and images for: Individual and population dietary specialization decline in fin whales during a period of ecosystem shift
Source: Sci Rep. 2021 Aug 25;11:17181. doi: 10.1038/s41598-021-96283-x (PMC8387503; doi:10.1038/s41598-021-96283-x)

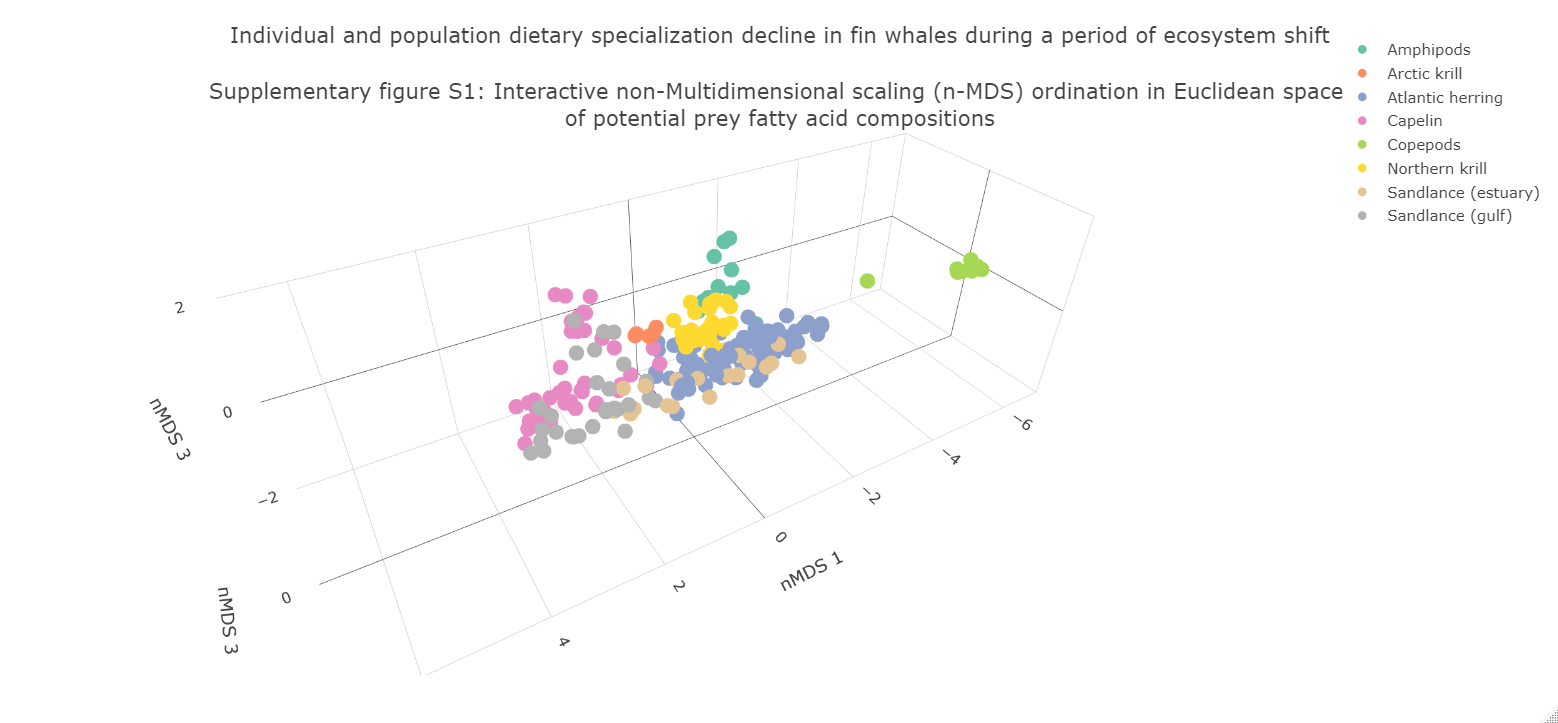

Supplement: Supplementary file 1 — Supplementary Information 1. [file 41598_2021_96283_MOESM1_ESM.png]

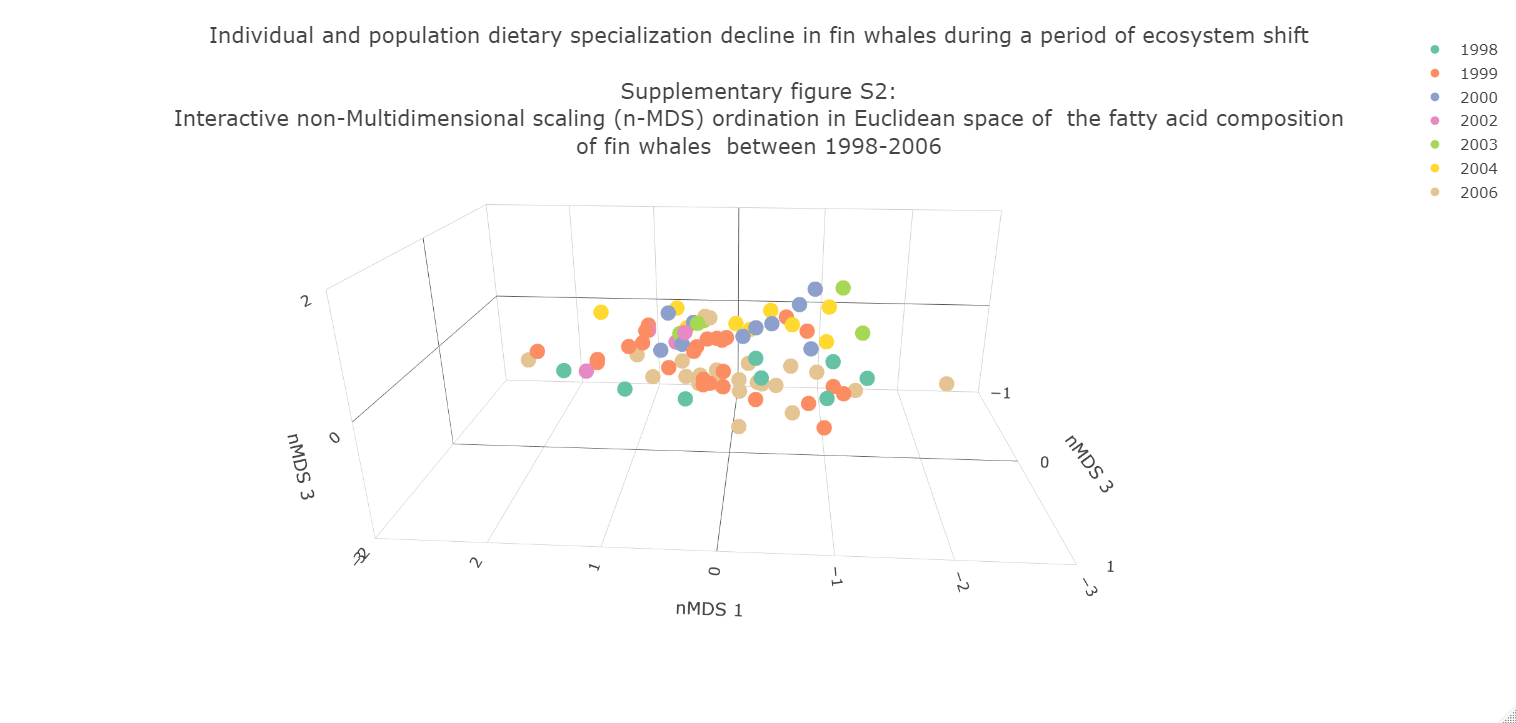

Supplement: Supplementary file 2 — Supplementary Information 2. [file 41598_2021_96283_MOESM2_ESM.png]
